# Improved functional and morphological outcomes with faricimab in nAMD eyes with poor response to prior intravitreal anti-VEGF therapy

**DOI:** 10.1007/s10792-025-03525-2

**Published:** 2025-05-08

**Authors:** Nathalie Bleidißel, Matthias Weichenberger, Mathias Maier, Nina Spielberg, Nikolaus Feucht

**Affiliations:** 1 Department of Ophthalmology, Klinikum Rechts Der Isar, Technical University Munich (TUM), Ismaningerstr. 22, 81675 Munich, Germany; 2 Smile Eyes Airport, Munich, Germany

**Keywords:** Neovascular age-related macular degeneration, Anti-VEGF, Faricimab, Poor treatment response, Spectral‐domain optical coherence tomography, Dosing interval

## Abstract

**Introduction:**

Neovascular age-related macular degeneration (nAMD) is a major cause of vision loss in older adults. While anti-VEGF therapies have improved management by suppressing abnormal blood vessel growth, a substantial subset of patients show poor functional as well as morphological responses and require frequent injections. Faricimab (Vabysmo®), a bispecific inhibitor targeting VEGF-A and angiopoietin-2 (Ang-2), has shown promise in achieving more durable disease control.

**Methods:**

This retrospective study included 48 eyes from 47 nAMD patients previously treated with ranibizumab or aflibercept, who were switched to faricimab due to poor treatment response. Evaluations occurred at four time points, assessing best-corrected visual acuity (BCVA), intraretinal (IRF) and subretinal fluid (SRF), subretinal pigment epithelium fluid and fibrosis, central subfield thickness (CST), and central subfield volume (CSV) using spectral-domain OCT. Dosing intervals and patient-reported outcomes were also recorded.

**Results:**

BCVA improved consistently, with mean logMAR improving from 0.54 to 0.40, reflecting a gain of 1.4 Snellen lines. Dosing intervals extended from a median of 5 to 8 weeks, with over one-third of eyes reaching intervals of 10 weeks or more. Significant reductions in IRF, SRF, CST as well as CSV were observed (p < 0.05) with a quarter of eyes showing no intra- or subretinal fluid at the fourth faricimab injection. Three patients were switched back to their previous anti-VEGF treatment due to a decline in BCVA.

**Discussion:**

The findings suggest Faricimab as an effective option for nAMD patients who respond inadequately to prior anti-VEGF therapies, offering both functional and anatomical improvements. Extended intervals reduce treatment burden, indicating faricimab’s potential to enhance disease control and patient quality of life in real-world settings.

## Introduction

Neovascular age-related macular degeneration (nAMD) remains a leading cause of blindness in western industrialized nations. In the affected population of older patients, this leads, often in stages, to severe visual impairment. Vascular endothelial growth factor (VEGF) plays an essential role in the pathogenesis of this disease, leading to abnormal formation of blood vessels in the retina and macula [1–3].

The introduction of anti-VEGF therapies, such as ranibizumab (Lucentis®, Novartis, Basel, Switzerland), aflibercept (Eylea®, Bayer, Leverkusen, Germany), brolucizumab (Beovu®, Novartis, Basel, Switzerland) and bevacizumab (Avastin®, Roche, Basel, Switzerland) off-label, leads to the suppression of pathological endothelial cell proliferation and vascular permeability and inhibits the development of choroidal or macular neovascularization (CNV/MNV). This has revolutionized the management of nAMD by slowing disease progression and improving the vision of many patients. Despite the success of these treatments, there remains a significant proportion of patients who show a limited response to these established anti-VEGF therapies. Such patients often require intensive monitoring and frequent injections, placing a significant burden on patients and the healthcare system [1–5]. With the development of faricimab (Vabysmo® Roche/Genentech, Basel, Switzerland), a novel bispecific inhibitor became available that inhibits both VEGF-A and angiopoietin-2 (Ang-2). Both molecules are essential in the pathogenesis of MNV membranes. By combined inhibition of both signaling pathways, faricimab may offer the potential to not only suppress VEGF-induced neovascularization but also reduce Ang-2-mediated vascular remodeling. This could potentially reduce disease activity and treatment frequency. In the controlled pivotial trial for the indication nAMD LUCERNE and TENAYA, faricimab was able to demonstrate efficient disease control by extending the injection interval after an upload phase of four monthly doses to up to 16 weeks without being inferior to aflibercept [6–8].

The aim of this study was to evaluate structural retinal biomarkers and function in eyes with nAMD after switching from an established VEGF inhibitor to faricimab. In addition to established biomarkers for disease activity, the analysis also focused on patients-reported functional results and treatment frequency. The results of this study could provide important insights into whether faricimab represents an effective alternative for patients inadequately responding to previous anti-VEGF therapies.

## Methods

### Study design

A single-center, retrospective analysis was conducted on consecutive patients with nAMD who were switched to faricimab due to poor response to prior treatment treatment with either aflibercept or ranibizumab. The study was carried out in the department of ophthalmology of the technical university of Munich and included patient records from the period October 2022 to April 2024. This study was approved by the local ethics committee and adhered to the tenets of the Declaration of Helsinki. All participants had given their written informed consent prior to the intravitreal injection. Medical records and spectral-domain optical coherence tomography (SD-OCT, Spectralis, Heidelberg) images were reviewed retrospectively.

### Data collection

For all patients, clinical data were collected at four key time points: at the first, second, third, and fourth intravitreal faricimab injection (IVT 1–4). The best-corrected visual acuity (BCVA) was assessed at each visit, alongside patient-reported subjective impression of visual function. SD-OCT imaging was performed at each visit to evaluate central subfield thickness (CST) in µm, central subfield volume (CSV) in mm^3^, intraretinal fluid (IRF), subretinal fluid (SRF) and subretinal pigment epithelium (SubRPE) complex (fluid or fibrosis). The intervals between injections were recorded. Additional covariates included patient age, gender, duration, number of prior anti-VEGF injections and lens status. Inclusion criteria were treatment with faricimab for nAMD and previous treatment with either aflibercept or ranibizumab with at least four injections. Only patients which were considered treatment-resistant due to no improvement in visual acuity, no morphological improvement or no elongation of dosing intervals were included. Exclusion criteria were eyes treated with less than three faricimab injections. The subtype of macular neovascularization (MNV) was determined for all included eyes based on fluorescein angiography. Cases of polypoidal choroidal vasculopathy were not included in the study.

### Treatment procedure

Patients were switched to faricimab based on specific criteria, including the presence of persistent IRF and SRF despite prior anti-VEGF therapy, progressive decline or lack of improvement in visual acuity and/or the need for short dosing intervals to maintain disease stability. These factors indicated a poor response to prior treatment, leading to a switch to faricimab. All patients received intravitreal faricimab injections either through a loading phase with injections every four weeks or with individualized dosing intervals based on their previous treatment regimen with ranibizumab or aflibercept. The decision to maintain prior dosing intervals rather than initiating a standardized loading phase was influenced by several factors. Some patients were unable to attend frequent clinic visits due to logistical constraints or poor overall health, making a strict upload strategy impractical. To ensure treatment adherence and maintain compliance, their previous injection intervals were therefore continued. Additionally, patients whose dosing intervals had already exceeded four weeks were unwilling to initiate a new therapy if it required another loading phase, despite the lack of improvement in visual acuity and/or retinal morphology under their prior therapy.

SD-OCT imaging and clinical evaluations were conducted prior to each injection, and dosing intervals were adjusted based on disease activity as determined by SD-OCT biomarkers and visual acuity. Patients with improved SD-OCT biomarkers (IRF, SRF, subRPE complex) were transitioned to extended dosing intervals by prolonging the prior interval by two weeks.

### Statistical analysis

Data analysis was performed using SPSS (version 28.0; SPSS Inc., Chicago, Illinois, USA). BCVA values were converted to the logarithm of the minimum angle of resolution (LogMAR) for statistical analysis. Continuous variables were reported as mean ± standard deviation (SD) or median and interquartile range, whereas categorical variables were expressed as percentages and absolutes. Comparisons between the time points were conducted using paired t-tests for continuous variables if normally distributed and McNemar-test or Wilcoxon-test were used for variables which were not normally distributed. Statistical significance was set at a p < 0.05 for all analyses.

## Results

After reviewing the medical records, 48 eyes from 47 patients were included in the analysis. The mean age was 78 (SD 8,5) years and 24 (51%) of the patients were male. The lens status of the patients remained stable over the four time points. 38 eyes (79.2%) were treated with aflibercept previously and ten eyes (20.8%) with ranibizumab. Each of the ten eyes treated with ranibizumab had already been treated with aflibercept in the past. 45 eyes (93.8%) continued with faricimab beyond our evaluation period. Only in three eyes (6.2%) a re-switch to ranibizumab or aflibercept was conducted during the course at the patient’s request due to a decline in BCVA and patient’s concerns regarding the efficacy of the novel treatment. No drug-related adverse events were reported, especially no intraocular inflammation (IOI). During the evaluation period, 48 eyes received at least three injections of faricimab, while 39 of those eyes received a fourth injection. Of the nine remaining eyes, six patients opted to continue treatment with their local ophthalmologist, three did not return for follow-up. Table 1 provides an overview of the functional and morphological parameters across the four time points.

**Table 1:** Characteristics of eyes over four faricimab injection timepoints

	IVT 1 (Switch)	IVT 2	IVT 3	IVT 4
Number of patients/eyes	47/48	47/48	47/48	39/39
Lens status, pseudophakic	33 (68.8%)	33 (68.8%)	33 (68.8%)	26 (66.7%)
BCVA (Mean LogMAR ± SD)	0.54 (± 0.45)	0.48 (± 0.46)	0.45 (± 0.38)	0.40 (± 0.31)
Intraretinal fluid	33 (68.8%)	22 (45.8%)	22 (46.8%)	21 (53.8%)
Subretinal fluid	31 (64.6%)	19 (39.6%)	19 (40.4%)	14 (35.9%)
Sub-RPE complex	41 (85.4%)	36 (75%)	35 (74.5%)	30 (76.9%)
Subretinal fluid maximum (in µm)	85.3 (± 77.2)	56.3 (± 90.0)	46.3 (± 66.9)	50.1 (± 72.2)
Central Subfield Volume (in mm^3^)	0.32 (± 0.12)	0.26 (± 0.11)	0.26 (± 0.10)	0.25 (± 0.08)
Central Subfield Thickness (in µm)	396.4 (± 149.0)	336.1 (± 138.2)	326.5 (± 117.0)	315.8 (± 96.5)

IVT = Intravitreal therapy; BCVA = best-corrected visual acuity; LogMAR = logarithm of minimal angle of resolution; SD = standard deviation; RPE = retinal pigment epithelium

### Visual acuity

**Fig. 1:**
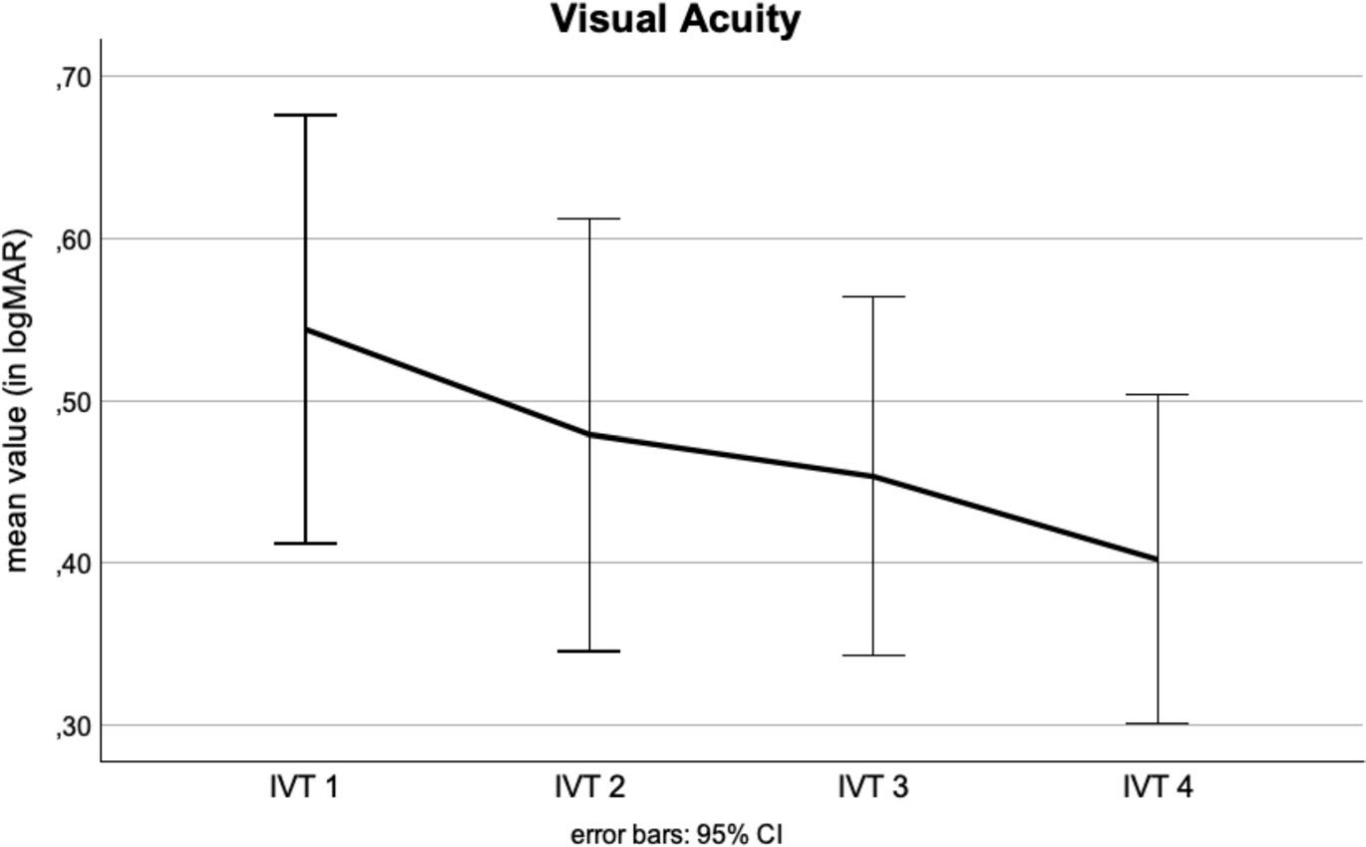
Mean visual acuity over the four injection timepoints. The graph illustrates the mean visual acuity (measured in logMAR) across four intravitreal faricimab injection (IVT) timepoints. It improved continuously from 0.54 (± 0.45), to 0.48 (± 0.46), to 0.45 (± 0.38), and finally to 0.40 (± 0.31) over the four time points (p < 0.05). Error bars represent the 95% confidence intervals for each timepoint

BCVA improved consistently in the study population, with mean logMAR values progressing from 0.54 (± 0.45) at baseline to 0.48 (± 0.46), then to 0.45 (± 0.38), and finally to 0.40 (± 0.31) over the four time points (p < 0.05, Fig. 1). This reflects an improvement of 1.4 Snellen lines. By the fourth faricimab injection, the mean visual acuity had reached a level sufficient for reading ability. 19 patients (39.6%) experienced an improvement in BCVA, 17 patients (35.4%) maintained stable BCVA, and 3 patients (6.2%) showed a decline in BCVA, leading to a re-switch to their prior anti-VEGF therapy. Subjectively, at the time of the switch, 43 patients (89.6%) reported an unchanged or worsened subjective impression of visual acuity. Following the switch to faricimab, 44 patients (91.7%) reported improved or unchanged subjective visual acuity compared to their previous medication.

No significant differences were observed between patients who experienced improvement in BCVA and those showed a stable or declined BCVA in terms of MNV type, age, prior therapy, or gender. Baseline characteristics were comparable across groups, suggesting that treatment response was not associated with these factors.

### Dosing intervals

**Fig. 2:**
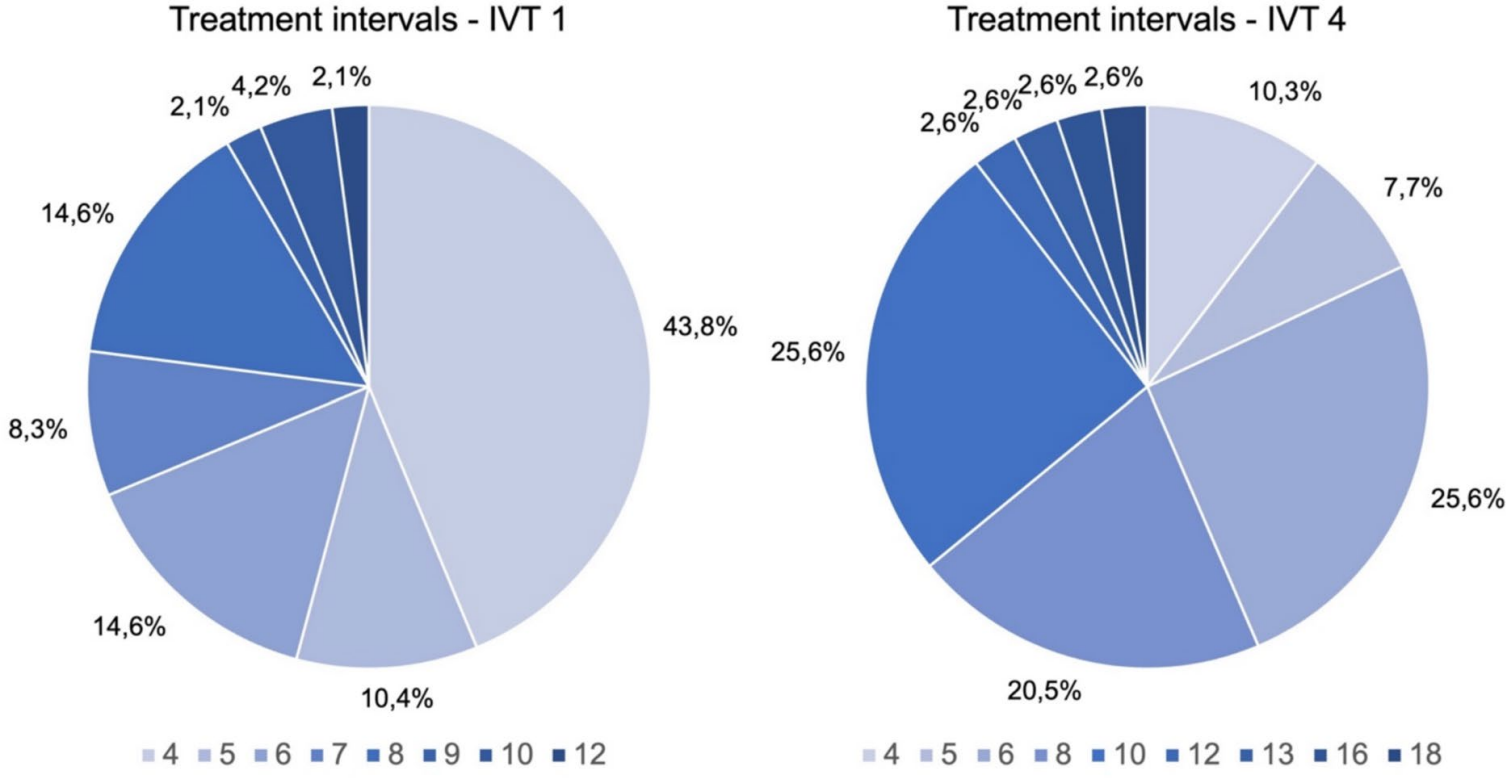
Distribution of Dosing Intervals at IVT 1 and IVT 4. The pie charts depict the distribution of dosing intervals in weeks at the first intravitreal faricimab injection (IVT 1) and the fourth intravitreal faricimab injection (IVT 4). At IVT 1, the majority of patients (43.8%) were on a 4-week interval. More than two-thirds of eyes (n = 33, 68,8%) were on an interval of 6 weeks or less. By the time of the fourth faricimab injection, 56,4% of eyes (n = 22) were on an 8-week or longer interval, and over one-third (n = 14, 36%) were on a 10-week or longer interval

At the time of the switch, over two-thirds of eyes (n = 33, 68.8%) were on an interval of 6 weeks or less. Notably, in 21 (43.8%) of the cases, an extension beyond a 4-week interval was not achieved under their previous therapy. At the time of the fourth faricimab injection, 22 eyes (56.4%) were on an 8-week or longer interval, and over one-third (n = 14, 36%) were on a 10-week or longer interval (Fig. 2). The median baseline interval was five weeks, which extended to eight weeks by the fourth faricimab injection, representing an average significant extension of three weeks (p < 0.001).

### Morphological outcomes

At all four time points, SD-OCT images were evaluated for the different compartments: IRF, SRF, and subRPE complex. The CST and CSV were also analyzed.

Upon switching to faricimab, IRF was present in 33 eyes (68.8%), while SRF was observed in 31 eyes (64.6%). IRF and SRF were simultaneously present in 18 eyes (37.5%), while only two eyes (4.2%) showed neither IRF nor SRF. These two cases were switched to faricimab due to persistently short dosing intervals of four weeks despite anatomical stability on OCT. Previous attempts to extend these intervals had led to the recurrence of SRF or IRF.

By the time of the fourth faricimab injection, 21 eyes (43.8%) had IRF and 14 eyes (29.2%) had SRF, with nine eyes (18.8%) showing both. A total of twelve eyes (25%) were considered dry, showing neither IRF nor SRF. Therefore, switching to faricimab led to a significant reduction in IRF as well as in SRF (p < 0.05).

Further, the maximum extend of SRF in the SD-OCT images was measured in the scan showing the most pronounced SRF. It decreased significantly over the course of measurements, from 85.3 µm to 50.1 µm by the time of the fourth faricimab injection (p < 0.05).

**Fig. 3:**
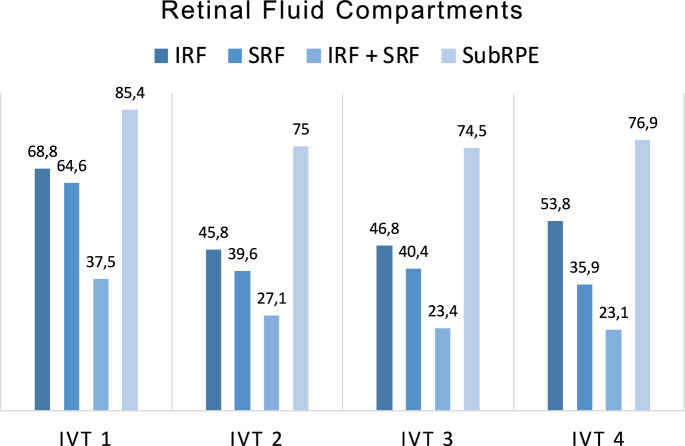
Retinal fluid compartments across four injection timepoints. The bar chart illustrates the presence of retinal fluid compartments and subretinal pigment epithelium (SubRPE) complex across four intravitreal faricimab injections (IVT). Intraretinal fluid (IRF) and subretinal fluid (SRF) show a consistent reduction over the course of treatment, with cases showing IRF and SRF decreasing from 18 eyes (37,5%) at IVT 1 to nine eyes (23,1%) at IVT 4 (p < 0.05). SubRPE complex was the most persistent feature, decreasing from 41 (85,4%) to 30 (62,5%), however the decrease was not significant (p > 0.05)

41 eyes (85.4%) showed subRPE complex at the time of the switch compared to 30 eyes (62.5%) at the time of the fourth faricimab injection (p > 0.05). These results indicate a reduction in all retinal fluid compartments with continued treatment, with only the subRPE complex remaining relatively stable (Fig. 3).

**Fig. 4:**
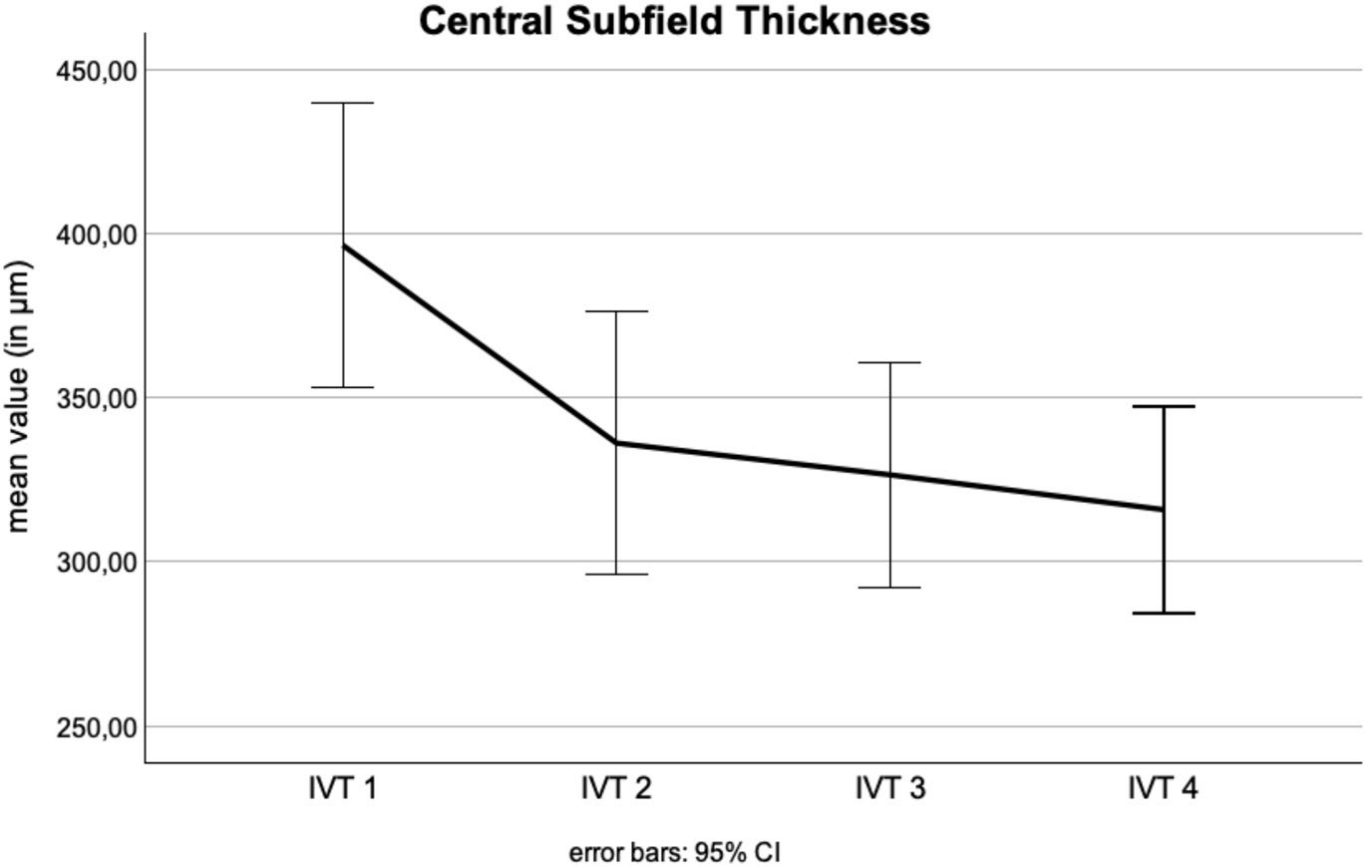
Central subfield thickness over four injection timepoints. The line graph shows the mean central subfield thickness (CST) in micrometers (µm) across four intravitreal faricimab injections (IVT 1–4), with error bars representing the 95% confidence intervals. A significant reduction in CST is observed from 396.4 µm (± 149.0), to 336.1 µm (± 138.2), to 326.5 µm (± 117.0), to 315.8 µm (± 96.5) over the course of treatment (p < 0.001)

The mean CST decreased significantly from 396.4 µm to 315.8 µm (p < 0.001, Fig. 4). Similarly, the mean CSV showed a significant reduction, from 0.32 mm^3^ to 0.25 mm^3^ (p < 0.001). CST and CSV showed moderate significant correlations with visual acuity across the four time points (r = 0.33–0.56, p < 0.05). Interestingly, higher CST and CSV values at the time of the switch were significantly correlated with poorer visual acuity outcomes even after 2, 3, and 4 faricimab injections (r = 0.36–0.56, p < 0.05). Thus, these findings suggest that initial CST and CSV can serve as predictors for visual acuity outcomes over the course of treatment.

The study cohort consisted of 27 eyes (56.3%) with type 1 MNV, 12 eyes (25%) with type 2 MNV, and 9 eyes (18.7%) with type 3 MNV. Subgroup analysis revealed no significant differences in treatment response in terms of BCVA or morphological changes among these MNV types in our study (p > 0.05).

## Discussion

The results of our study align with growing evidence supporting faricimab as an effective option for managing nAMD, particularly in eyes with poor responses to prior anti-VEGF therapies. Key outcomes – such as improved visual acuity, extended dosing intervals, and consistent reductions in CSV, CST, IRF, and SRF – underscore its functional and morphological benefits. These findings build on pivotal trials like TENAYA and LUCERNE, which demonstrated faricimab’s non-inferior efficacy to aflibercept, alongside the advantage of prolonged dosing intervals [6–8]. Moreover, faricimab has been shown to have a favorable safety profile, and while some studies have reported cases of IOI, we did not observe any instances of IOI in our cohort [24–28]. By demonstrating its real-world effectiveness in eyes with poor response to prior anti-VEGF therapies, our study highlights faricimab’s potential to reduce both patient burden and healthcare resource demands.

### Improvement in visual acuity

In our study, visual function improved significantly, with mean visual acuity reaching levels generally compatible with reading ability. This contrasts with other studies, which largely reported visual stabilization rather than improvement after switching to faricimab, despite clear anatomical benefits [9–13, 22]. However, our findings, along with two other real-world studies, suggest that non-naïve patients who responded poorly to prior anti-VEGF therapy may still achieve functional improvement after a switch to faricimab [14, 15]. Additionally, we observed a significant enhancement in patient-reported subjective impression of visual acuity compared to their previous treatment. To our knowledge, this is the first study to include patient-reported subjective impression of visual function. Okanda and colleagues identified lack of motivation as a key factor in treatment non-adherence [16]. The positive subjective feedback following the switch to faricimab highlights improved patient satisfaction, which may support better long-term adherence.

### Extension of dosing intervals

In our study, the extension of dosing intervals following the switch to faricimab underscores one of its main advantages in managing nAMD patients who responded poorly to prior anti-VEGF therapies. At the time of the switch, over two thirds of patients required injections every four to six weeks. By the fourth faricimab injection, over half of the patients achieved intervals of eight weeks or longer, with more than a third extending to ten weeks or beyond. This reduces the burden of frequent clinic visits, improving patient adherence and quality of life [16]. The dual VEGF-A and Ang-2 inhibition targets both neovascularization and vascular instability, likely contributing to stable disease control and prolonged intervals [17]. Our findings align with the TENAYA and LUCERNE trials, where faricimab demonstrated prolonged dosing intervals while maintaining efficacy [6–8]. However, these trials were conducted on therapy-naïve eyes. Real-world studies from Japan, the UK, and Switzerland, reported similar interval extensions in refractory nAMD cases, as observed in our study [9–12]. Prolonging dosing intervals without compromising retinal stability offers significant clinical value, and future research could identify patient factors predicting successful extension for more personalized treatment.

### Reduction in central subfield volume (CSV) and central subfield thickness (CST)

Our study demonstrated a significant reduction in CSV and CST, which correlated strongly with visual acuity improvement across all four time points, highlighting these biomarkers as potential prognostic factors for long-term outcomes. Similarly, other studies reported a reduction in CST [9–11, 14, 15, 18, 19]. Notably, we are the first to identify initial CST at the time of the switch as a biomarker for visual acuity during faricimab treatment. The robust reduction in macular edema observed, potentially due to additional Ang-2 inhibition and improved vascular stability, underscores faricimab's potential to deliver consistent anatomical and functional improvements, particularly in nAMD patients with chronic fluid accumulation and poor response to prior anti-VEGF therapy.

### Reduction in intraretinal fluid (IRF) and subretinal fluid (SRF)

Our study demonstrated a significant reduction in IRF and SRF, with a quarter of eyes achieving a dry macula by the fourth faricimab injection Importantly, these improvements were achieved in a cohort of patients previously considered to be poor responders to anti-VEGF therapy. Our findings align with other studies reporting 27%−32% of eyes achieving a dry macula after switching to faricimab, further supporting that eyes with insufficient response to prior anti-VEGF therapies show improved IRF and SRF resolution with faricimab [9, 15, 19–22]. These results highlight faricimab’s efficacy in managing persistent fluid, a common challenge in eyes with poor response to anti-VEGF-treatment, and its potential to stabilize retinal function while preventing further anatomical damage. Interestingly, this potential effect was also discussed in a recent publication demonstrating that switching from aflibercept to faricimab reduces Ang-2 levels and suggesting that faricimab’s dual-target mechanism effectively addresses exudative changes by suppressing Ang-2 expression [23].

### Strengths and limitations

Our study has several strengths. First, by including eyes previously treated with anti-VEGF therapies but showing limited response, we evaluated faricimab’s effectiveness in a challenging real-world population, which better reflects clinical practice. Second, unlike clinical trials, this study allowed flexible dosing intervals from the outset, as some patients opted out of an initial upload phase due to personal or logistical reasons. This flexibility offers insights into faricimab's performance under less intensive early treatment, providing a practical perspective on real-world treatment adherence and its challenges. This variation in treatment approach could have led to an underestimation of faricimab’s full potential, as patients who did not receive a loading phase may have experienced a less pronounced initial response. Consequently, our findings are more likely to be biased toward conservative estimates of efficacy rather than overstating the treatment’s benefits. This is particularly noteworthy as it reflects the settings most commonly encountered by clinicians in real-world practice. Further, we included the patient-reported subjective impression of visual function, which represents a factor not to be underestimated in the clinical setting. Finally, the use of standardized SD-OCT analysis strengthens the study's reliability, offering a precise assessment of morphological outcomes.

However, our study has several limitations. All participants had previously received anti-VEGF therapy, which may have influenced their responses to faricimab, limiting generalizability to treatment-naïve eyes. Second, the absence of an initial loading phase may have impacted the observed functional and morphological outcomes. These outcomes might have been even more pronounced if a loading phase had been implemented for each eye. Third, the qualitative nature of patient-reported subjective impressions, which were documented in the medical records as ‘improved,’ ‘unchanged,’ or ‘worsened’ without the use of a standardized questionnaire limits the granularity of subjective assessments. Fourth, while CST and CSV were used as primary anatomical outcome measures in our study, we acknowledge that more advanced volumetric assessments of IRF, SRF, and PED could provide a more detailed understanding of anatomical changes. Such methods allow for a more precise evaluation of disease activity and treatment response, potentially capturing subtle morphological variations that may not be reflected in CST and CSV alone [29]. Lastly, the relatively short follow-up period limits our ability to assess faricimab’s durability in sustaining morphological and functional improvements over time. Future research with extended follow-up and inclusion of treatment-naïve eyes as well as nAMD-subtype analysis will provide a more comprehensive understanding of faricimab’s role in nAMD management.

## Conclusion

Our study supports faricimab as a promising treatment option for nAMD, particularly in patients with poor responses to prior anti-VEGF therapies. Significant and continuous improvements in visual acuity, patient-reported subjective impression of visual function, and anatomical outcomes, including reductions in IRF, SRF, CST, and CSV, were observed. Extended dosing intervals, with over half of patients achieving eight weeks or longer by the fourth injection, highlight its potential to reduce treatment burden. The dual VEGF and Ang-2 inhibition potentially drives these functional and morphological benefits, making faricimab effective for managing persistent fluid and stabilizing retinal function in therapy resistant cases. Initial CST emerged as a potential biomarker for visual acuity outcomes. Further research on retinal biomarkers and patient-specific factors, such as phenotype and fluid chronicity, will be crucial in optimizing faricimab’s application.

## Data Availability

All the data are available upon request. The data that support the findings of this study are not openly available due to reasons of sensitivity and are available from the corresponding author upon reasonable request.
